# Platelet-to-Albumin Ratio: A Novel IgA Nephropathy Prognosis Predictor

**DOI:** 10.3389/fimmu.2022.842362

**Published:** 2022-05-19

**Authors:** Jiaxing Tan, Guojiao Song, Siqing Wang, Lingqiu Dong, Xiang Liu, Zheng Jiang, Aiya Qin, Yi Tang, Wei Qin

**Affiliations:** ^1^ Division of Nephrology, Department of Medicine, West China Hospital, Sichuan University, Chengdu, China; ^2^ West China School of Medicine, Sichuan University, Chengdu, China

**Keywords:** platelet-to-albumin ratio, IgA nephropathy, prognosis, cohort study, end-stage renal desease

## Abstract

**Background:**

Chronic inflammation is related to the development of IgA nephropathy (IgAN). Emerging studies have reported that platelet-related parameters including platelet (PLT), platelet-to-albumin ratio (PAR), and platelet-to-lymphocyte ratio (PLR) are proved to be novel prognostic indicators for several inflammatory diseases. Whether platelet-related parameters could serve as predictors for IgAN remains unknown.

**Methods:**

A total of 966 IgAN patients were enrolled in this retrospective study and were divided into several groups based on the optimal cut-off value of the platelet-related parameters. End-stage renal disease was used as the renal endpoint. A 1:2 propensity score (PS) match was then carried out to eliminate significant differences at baseline. The area under the receiver operating characteristic curve (AUROC), Kaplan–Meier (K-M) curve, and Cox proportional hazards analyses were performed to evaluate their predictive effect.

**Results:**

Without considering the effect of covariates, the K-M curve showed that PLT, PLR, and PAR were strongly correlated with the renal outcomes of IgAN. However, the AUROC revealed that the PAR and PLR had better predictive power than the PLT. Multivariate Cox regression adjusting for demographic data, pathological findings, treatment, and laboratory results indicated that compared with PLR, albumin and PLT, PAR seemed to be a better marker of adverse renal outcome, implying that PAR was the only platelet-related parameter that could be used as an independent risk factor. Notably, high PAR patients seemed to have more severe clinical manifestations and pathological lesions. However, after eliminating the influence of different baselines on outcome variables, the PAR could still predict the poor prognosis of IgAN. To more accurately evaluate the predictive power of the PAR, we analyzed the predictive effect of the PAR on patients with different clinicopathological characteristics through subgroup analysis. It was indicated that the PAR might better predict the prognosis and outcome of patients whose disease was already very severe.

**Conclusion:**

PAR might be used as an independent risk factor for IgAN progression.

## Introduction

IgA nephropathy (IgAN) is the most common type of primary glomerulonephritis worldwide and approximately 30-40% of IgAN patients may lose their kidney function gradually and progress to end-stage renal disease (ESRD) within 20 years from the time of diagnosis. Patients are miserable and bear a huge financial burden due to the high recurrence rates and poor renal prognosis of IgAN (1). Therefore, it is particularly important to identify high-risk patients and take corresponding measures. Accumulating evidence has illustrated that IgAN is an immune system disease, where inflammation is closely related to the severity and prognosis of the disease (2).

Platelet-related parameters, including platelet (PLT), platelet-to-albumin ratio (PAR), and platelet-to-lymphocyte ratio (PLR) are easy to obtain clinically and have been proved to be novel prognostic indicators for several inflammatory diseases (3–5). In addition to hemostasis, platelets may also be able to trigger and aggravate inflammation by interacting with immune cells and secreting proinflammatory cytokines (6). Moreover, the results of multiple studies consistently report that PAR and PLR are associated with inflammation and have been described as emerging inflammation indexes (3–5). Nevertheless, few studies have demonstrated the precise relationship between platelet-related parameters and IgAN. Accordingly, we examined the data of 966 patients with IgAN to determine whether platelet-related parameters are risk factors for ESRD in patients with IgAN.

## Methods

### Patient Selection

The study included 1,570 patients with IgAN diagnosed by renal biopsy at West China Hospital of Sichuan University between January 2009 and December 2018. Among these, 209 patients without sufficient pathologic data or renal biopsies containing fewer than eight glomeruli, 303 individuals whose data were missing during follow-up, and 27 subjects with less than 12 months of follow-up before reaching the endpoint were excluded from the study. In addition, we excluded 23 patients with secondary IgAN and 22 patients with active infection. Considering that prednisone or other immunosuppressive treatments might impact the level of platelet-related parameters, a total of 20 patients who received such therapy before renal biopsy were excluded from this study. Ultimately, 966 patients were enrolled in our following study. All patients were followed up in the outpatient clinic at least every 1-3 months after the kidney biopsy. The study was approved by the Ethics Committee of West China Hospital of Sichuan University (2019-33), and all methods were carried out according to relevant guidelines and regulations. All patients signed a written informed consent form and agreed to participate in this study.

### Clinical and Pathological Data Collection

At the time of renal biopsy and the follow-up visit, demographic information, clinical data and experimental data were gathered. Hypertension was defined as SBP≥140 mmHg and/or DBP≥90 mmHg at rest. The Oxford classification (M, mesangial hypercelluarity; S, glomerulosclerosis; E, endocapillary hypercellularity; T, tubular atrophy and interstitial fibrosis; and C, cellular or fibro-cellular crescents) was used to evaluate the pathological lesions (7). Anemia was defined as a hemoglobin concentration lower than 120 g/L in men or lower than 110 g/L in women. Hypoalbuminemia was defined as albumin < 30 g/L.

### Treatment and Renal Endpoint

The treatment was mainly based on KDIGO guidelines, which were finally determined by the attending doctors and the patients (8). The treatment therapies included adequate doses of renin-angiotensin-aldosterone system blockers, the standard-dose glucocorticoid regimen and other immunosuppressants. Notably, the latter two were collectively referred to as immunosuppressive therapy (IST).

ESRD was used as a renal endpoint, which was defined as an estimated glomerular filtration rate (eGFR) less than 15 mL/min/1.73 m^2^ and/or the start of renal replacement therapy (9).

### The Definition and Predictive Value of Platelet-Related Parameters

PLT was obtained by the absolute platelet count of routine blood examination, where platelets were measured per microliter of blood. The absolute platelet count divided by the serum albumin level was PAR. The ratio of the absolute number of platelets to lymphocytes was the PLR.

The receiver operating characteristic curve (AUROC) was utilized to supervise the power to discriminate various predictive factors for progression of ESRD. The optimal cut-off points of the PAR, PLR, and PLT were scored by the Youden index (sensitivity + specificity – 1) (10). The cut-off value was considered to be the optimal value in this study when PAR, PLR, and PLT corresponded to the maximum Youden index.

### Statistical Analysis

Data were analyzed using Microsoft Excel and SPSS software. Univariate analysis, followed by multivariate linear regression, was used to determine independent predictors or risk factors. The relationship between parameters and renal survival was assessed through Cox regression. Normally continuous variables are expressed as the means ± SD and were compared using a T test. P<0.05 were considered statistically significant. Nonparametric variables are usually expressed as medians with interquartile ranges and were compared using either the Mann-Whitney U or Kruskal-Wallis test. Categorical variables were compared using a χ^2^ test.

A 1:2 propensity score (PS) match was then carried out to eliminate significant differences at baseline. All the variables that were different at baseline were considered as covariates. According to the greedy matching algorithm, multi-logit regression was performed to make all the parameters comparable at baseline (11, 12). The new cohort performed subsequent statistics in the same way as above.

## Results

### Patients

A total of 1,617 patients with IgAN were involved while 604 individuals were excluded for the following reasons: secondary IgAN, less than 12 months of follow-up before reaching endpoints, active infection, prednisone or other immunosuppressive treatment before renal biopsy, or insufficient pathological data. After strict inclusion and exclusion criteria, 966 patients were finally included in this study. The individuals with a mean age of 34.52 ± 11.30 years old were followed up for 58.67 ± 28.53 months on average. Notably, 61 patients progressed to ESRD, accounting for 6.3% of the total number.

### Predictive Value of Platelet-Related Parameters for ESRD

**Figure 1:**
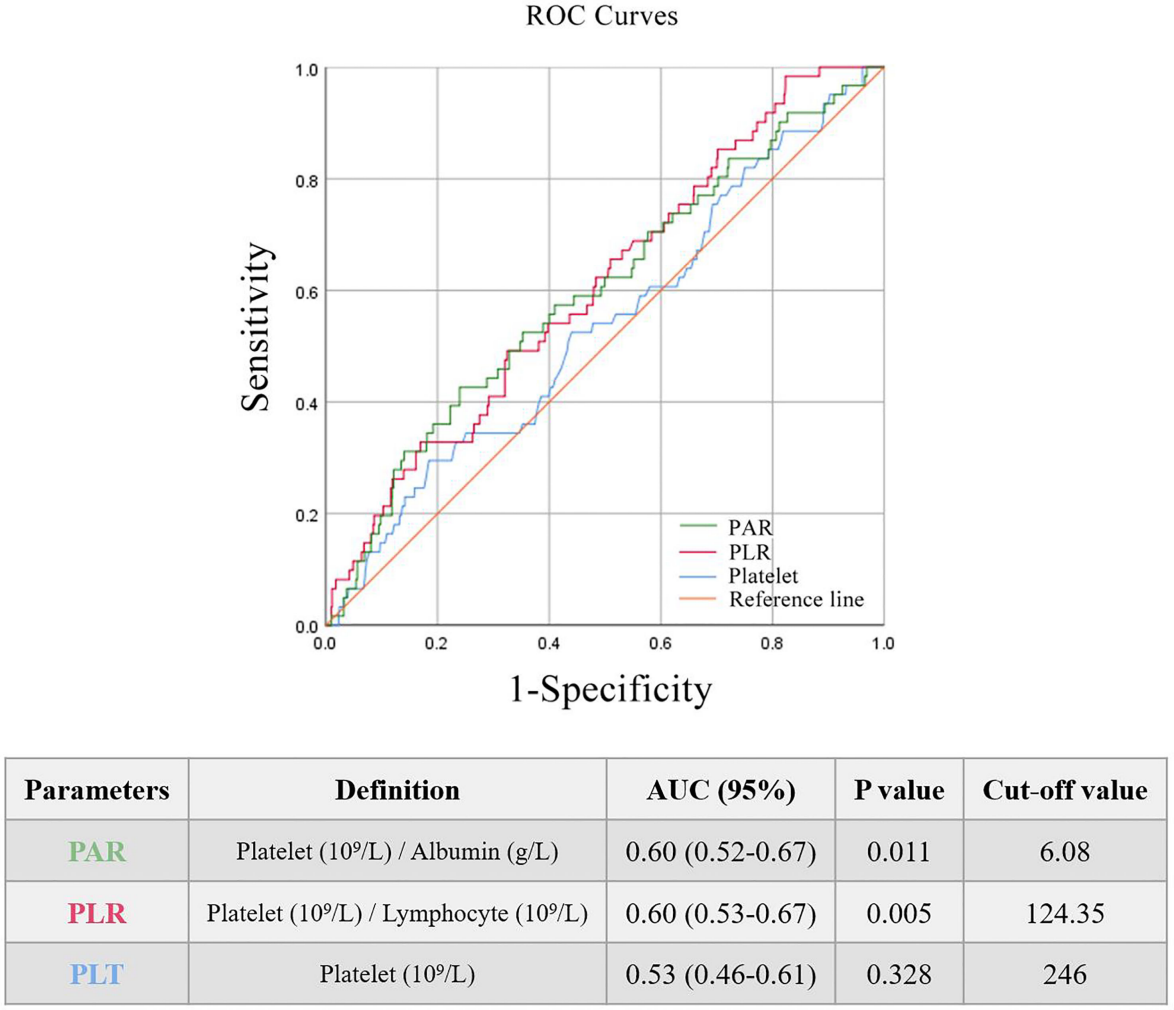
The areas under the ROC curves of platelet-related parameters.

The AUROC was used to identify the prognostic value of the indictors related to platelets. Three main parameters (PAR, PLR, and PLT) were compared and all of them seemed to be associated with renal outcomes of IgAN. It was found that PAR and PLR had better predictive power (**Figure 1**) than PLT.

**Figure 2:**
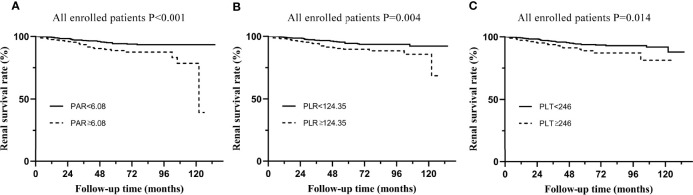
Kaplan-Meier analysis for the endpoint of ESRD in all enrolled patients. **(A)** Patients were divided by PAR. **(B)** Patients were divided by PLR. **(C)** Patients were divided by PLT.

All patients enrolled in the study were divided into several subgroups according to their PAR, PLR, and PLT at the time of renal biopsy to investigate the relationship between platelet-related parameters and prognosis. The optimal cut-off values of PAR, PLR, and PLT were 6.08, 124.35 and 246, respectively, which was mainly based on the Youden index. There were remarkable differences in renal survival rates between patients with various degrees of PAR, with 26/243 (10.7%) and 35/723 (4.8%) in the high PAR and low PAR groups, respectively (**Figure 2A**). In addition, it was found that individuals with high PLR or PLT usually had worse clinical outcomes than patients in the low PLR or PLT group (P<0.05, **Figures 2B, C**).

### PAR as an Independent Risk Factor for the Progression of IgAN to ESRD

The renal endpoint in this study was ESRD. A multivariate Cox regression model was used to identify independent risk factors for IgAN (**Tables 1**, **2**). The results of Model 1, including demographics, clinicopathological features, and PAR indicated that PAR was an independent risk factor for ESRD (HR 3.35, 95% CI 1.38-8.14, P=0.008, **Table 1**). Model 2 added PLR and PLT on the basis of model 1. Although the results of univariate analysis showed that PAR (HR 2.43, 95% CI 1.46-4.04, P=0.001), PLR (HR 2.05, 95% CI 1.24-3.39, P=0.005), and PLT (HR 1.97, 95% CI 1.13-3.42, P=0.016) were related to renal progression and multivariate Cox regression analysis demonstrated that only PAR was a risk factor affecting the prognosis of the kidney, while PLR and PLT were not (**Table 3**). In addition, immunosuppressive therapy, tubular atrophy or interstitial fibrosis, CKD stages, and anemia might also serve as prognostic factors of IgAN.

**Table 1:** Prediction of renal outcomes in the IgAN carried out by Cox-regression analysis adjusted for PAR, clinicopathologic findings and demographic data.

	Univariate analysis		Multivariate analysis	
	HR (95% CI)	p	HR (95% CI)	p
**High PAR (vs. low PAR)**	2.43 (1.46-4.04)	**0.001**	3.35 (1.38-8.14)	**0.008**
Age (years)	1.00 (0.98-1.02)	0.993	0.98 (0.95-1.01)	0.109
Male (vs. Female)	2.23 (1.32-3.76)	**0.003**	1.68 (0.82-3.43)	0.157
Smoking	2.06 (1.19-3.57)	**0.010**	1.38 (0.62-3.07)	0.435
Drinking	1.25 (0.71-2.22)	0.439	0.49 (0.24-1.01)	0.054
Hypertension	3.93 (2.36-6.55)	**<0.001**	1.25 (0.68-2.29)	0.468
Immunosuppressive therapy	0.76 (0.46-1.25)	0.280	0.34 (0.19-0.61)	**<0.001**
**Oxford Classification**				
M1 (vs. M0)	4.51 (1.64-12.50)	**0.004**	2.51 (0.88-7.13)	0.085
E1 (vs. E0)	1.93 (0.77-4.81)	0.160	1.00 (0.35-2.88)	0.999
S1 (vs. S0)	2.82 (1.53-5.23)	**0.001**	1.50 (0.72-3.14)	0.277
T1/T2 (vs. S0)	14.52 (8.01-26.04)	**<0.001**	2.33 (1.12-4.84)	**0.024**
C1/C2 (vs. C0)	1.92 (1.15-3.21)	**0.013**	1.17 (0.65-2.11)	0.598
**Laboratory findings**				
Proteinuria (g/24h)	1.11 (1.06-1.17)	**<0.001**	1.08 (0.96-1.21)	0.186
U-RBC (/HP)	0.999 (0.998-1.001)	0.496	0.999 (0.998-1.003)	0.471
CKD stages				
Stage 2 (vs. Stage 1)	4.79 (1.24-18.53)	**0.023**	3.48 (0.85-14.30)	0.084
Stage 3/4 (vs. Stage 1)	49.75 (15.52-159.46)	**<0.001**	19.01 (4.87-74.94)	**<0.001**
Anemia	4.29 (2.54-7.24)	**<0.001**	2.28 (1.24-4.19)	**0.008**
Hypoalbuminemia (≤30g/L)	2.04 (1.06-3.92)	**0.032**	0.71 (0.29-1.74)	0.452
PLT (×10^9^/L)	1.002 (0.999-1.006)	0.220	0.997 (0.992-1.003)	0.397

PAR, platelet to albumin ratio; M, mesangial proliferation; E, endocapillary proliferation; S, segmental glomerulosclerosis; T, tubular atrophy or interstitial fibrosis; C, crescents; U-RBC, the count of uric red blood cell; PLT, platelet.

Bold values was that the differences were significant.

**Table 2:** Prediction of renal outcomes in the IgAN carried out by Cox-regression analysis adjusted for PAR, PLR, clinicopathologic findings and demographic data.

	Univariate analysis		Multivariate analysis	
	HR (95% CI)	p	HR (95% CI)	p
**High PAR (vs. low PAR)**	2.43 (1.46-4.04)	**0.001**	2.62 (1.06-6.49)	**0.037**
**High PLR (vs. low PLR)**	2.05 (1.24-3.39)	**0.005**	0.92 (0.51-1.68)	0.786
**High PLT (vs. low PLT)**	1.97 (1.13-3.42)	**0.016**	1.05 (0.43-2.58)	0.923
Age (years)	1.00 (0.98-1.02)	0.993	0.98 (0.95-1.01)	0.123
Male (vs. Female)	2.23 (1.32-3.76)	**0.003**	1.67 (0.81-3.47)	0.166
Smoking	2.06 (1.19-3.57)	**0.010**	1.35 (0.59-3.08)	0.477
Drinking	1.25 (0.71-2.22)	0.439	0.50 (0.24-1.03)	0.061
Hypertension	3.93 (2.36-6.55)	**<0.001**	1.26 (0.68-2.33)	0.457
Immunosuppressive therapy	0.76 (0.46-1.25)	0.280	0.35 (0.19-0.62)	**<0.001**
**Oxford Classification**				
M1 (vs. M0)	4.51 (1.64-12.50)	**0.004**	2.55 (0.90-7.26)	0.079
E1 (vs. E0)	1.93 (0.77-4.81)	0.160	0.98 (0.34-2.82)	0.970
S1 (vs. S0)	2.82 (1.53-5.23)	**0.001**	1.45 (0.70-3.03)	0.318
T1/T2 (vs. S0)	14.52 (8.01-26.04)	**<0.001**	2.39 (1.15-4.99)	**0.020**
C1/C2 (vs. C0)	1.92 (1.15-3.21)	**0.013**	1.16 (0.64-2.09)	0.633
**Laboratory findings**				
Proteinuria (g/24 h)	1.11 (1.06-1.17)	**<0.001**	1.07 (0.95-1.20)	0.299
U-RBC (/HP)	0.999 (0.998-1.001)	0.496	0.999 (0.997-1.001)	0.439
CKD stages				
Stage 2 (vs. Stage 1)	4.79 (1.24-18.53)	**0.023**	3.48 (0.85-14.28)	0.083
Stage 3/4 (vs. Stage 1)	49.75 (15.52-159.46)	**<0.001**	19.15 (4.89-74.96)	**<0.001**
Anemia	4.29 (2.54-7.24)	**<0.001**	2.36 (1.28-4.34)	**0.006**
Hypoalbuminemia (≤30 g/L)	2.04 (1.06-3.92)	**0.032**	0.81 (0.33-1.98)	0.640

PAR, platelet to albumin ratio; PLR, platelet to lymphocyte ratio; PLT, platelet; M, mesangial proliferation; E, endocapillary proliferation; S, segmental glomerulosclerosis; T, tubular atrophy or interstitial fibrosis; C, crescents; U-RBC, the count of uric red blood cell.

Bold values was that the differences were significant.

**Table 3:** Clinicopathological manifestations of the IgAN patients at baseline, grouped by the platelet to albumin ratio.

Variables	Unmatched Cohort			Matched Cohort		
	Low PAR group	High PAR group	P	Low PAR group	High PAR group	P
Numbers (%)	723 (74.8)	243 (25.2)		115 (66.9)	57 (33.1)	
Age at diagnosis (years)	33 (26-42)	33 (24-43)	0.808	35.2 ± 11.5	33.2 ± 12.1	0.292
Male (%)	343 (47.4)	100 (41.2)	0.102	53 (46.1)	22 (38.6)	0.415
Smoking (%)	126 (17.4)	46 (18.9)	0.628	18 (15.7)	8 (14.0)	0.826
Drinking (%)	156 (21.6)	54 (22.2)	0.857	19 (16.5)	10 (17.5)	1.000
SBP (mmHg)	125 (115-137)	124 (114-140)	0.779	127.9 ± 18.3	127.8 ± 18.2	0.957
DBP (mmHg)	83.1 ± 13.2	83.4 ± 14.3	0.731	82.8 ± 14.0	83.1 ± 16.6	0.891
Hypertension (%)	186 (25.7)	71 (29.2)	0.314	31 (27.0)	11 (19.3)	0.346
Proteinuria (g/24 h)	1.10 (0.66-2.11)	2.53 (1.29-5.8)	**<0.001**	2.00 (1.00-3.00)	2.00 (1.00-3.00)	0.337
U-RBC (/HP)	18 (6-63)	22 (7-80)	0.197	23 (6-80)	24 (7-71)	0.090
PLT (×10^9^/L)	172 (137-201)	265 (232-301)	**<0.001**	170.5 ± 45.7	277.8 ± 59.5	**<0.001**
ALB (g/L)	41.4 (38.4-44.2)	35.4 (28.3-39.5)	**<0.001**	40.1 (37.1-43.3)	36.4 (31.6-40.8)	**<0.001**
e-GFR (ml/min/1.73m^2^)	93.03 (67.50-116.56)	94.00 (60.13-134.26)	0.593	83.82 ± 32.09	93.06 ± 37.86	0.096
sCr (umol/L)	83.0 (66.0-108.4)	81.9 (62.0-112.0)	0.227	103.1 ± 48.5	95.8 ± 56.6	0.375
Hb (g/L)	133 (120-148)	132 (117-145)	0.217	131.4 ± 21.5	127.2 ± 20.7	0.222
Anemia (%)	216 (29.9)	75 (30.9)	0.809	39 (33.9)	21 (36.8)	0.376
M0/M1 (%)	183/540 (25.3/74.7)	52/191 (21.4/78.6)	0.228	34/81 (29.6/70.4)	10/47 (17.5/82.5)	0.098
E0/E1 (%)	703/20 (97.2/2.8)	222/21 (91.4/8.6)	**<0.001**	115/0 (100/0)	56/1 (99.4/1.8)	0.331
S0/S1 (%)	279/444 (38.6/61.4)	93/150 (38.3/61.7)	0.939	35/80 (30.4/69.6)	22/35 (38.6/61.4)	0.350
T0/T1-2 (%)	586/137 (81.1/18.9)	185/58 (76.1/23.9)	0.116	86/29 (74.8/25.2)	42/15 (73.7/26.3)	1.000
C0/C1-2 (%)	564/159 (78.0/22.0)	173/70 (71.2/28.8)	**0.036**	81/34 (70.4/29.6)	37/20 64.9/35.1)	0.488
Immunosuppressive therapy	387 (53.5)	182 (74.9)	**<0.001**	75 (65.2)	39 (68.3)	0.734

SBP, systolic blood pressure; DBP, diastolic blood pressure; U-RBC, the count of uric red blood cell; ALB, albumin; sCr, serum creatinine; e-GFR, estimated glomerular filtration rate; Hb, hemoglobin; PLT, platelet; M, mesangial proliferation; E, endocapillary proliferation; S, segmental glomerulosclerosis; C, crescents; T, tubular atrophy/interstitial fibrosis.

Bold values was that the differences were significant.

### Clinicopathological Features of Patients With Different Levels of PAR

From the above statistical results, it was not difficult to see that the PAR was the most significant prognostic indicator among all platelet-related parameters. Therefore, we divided the patients into two groups according to the best cut-off point of PAR, and **Table 3** presents the baseline clinicopathological characteristics of the two groups. The optimal cut-off value of PAR was 6.08. A total of 723 patients (343 men) were categorized into the low PAR group, while 243 patients (100 men) were assigned to the high PAR group. Apart from hypoalbuminemia and high levels of platelets, patients with high levels of PAR tended to have more severe proteinuria, more obvious pathological lesions (endocapillary proliferation and crescents), and more aggressive treatment. No significant differences were observed in age, sex, blood pressure, smoking, drinking, initial renal function, or anemia between the two groups. These results indicated that the PAR index might be related to clinical parameters and pathologic lesions of IgAN.

Considering that platelets usually interacted with platelets express receptors, notably the CD32A, an IgG Fc receptor, we have supplemented the data on the relationship between the PAR and autoimmune antibodies, which is presented in the supplementary **Table 1**. Interestingly, there was a correlation between the PAR and IgG. In the unmatched cohort, patients in the low PAR group tended to have a higher level of IgG levels. The levels of IgM, C3, and C4 in the low PAR group were slightly lower than those in the high PAR group. After PS matching, the differences in IgM, C3, and C4 levels disappeared while low PAR group still had a higher level of IgG.

The data that compared the value of PAR at the time of renal biopsy and the last follow-up is presented in the supplementary **Table 2**. We found that there was no significant difference between two treatment groups about the change value of PAR.

### Use of PAR to Predict Renal Prognosis

**Figure 3:**
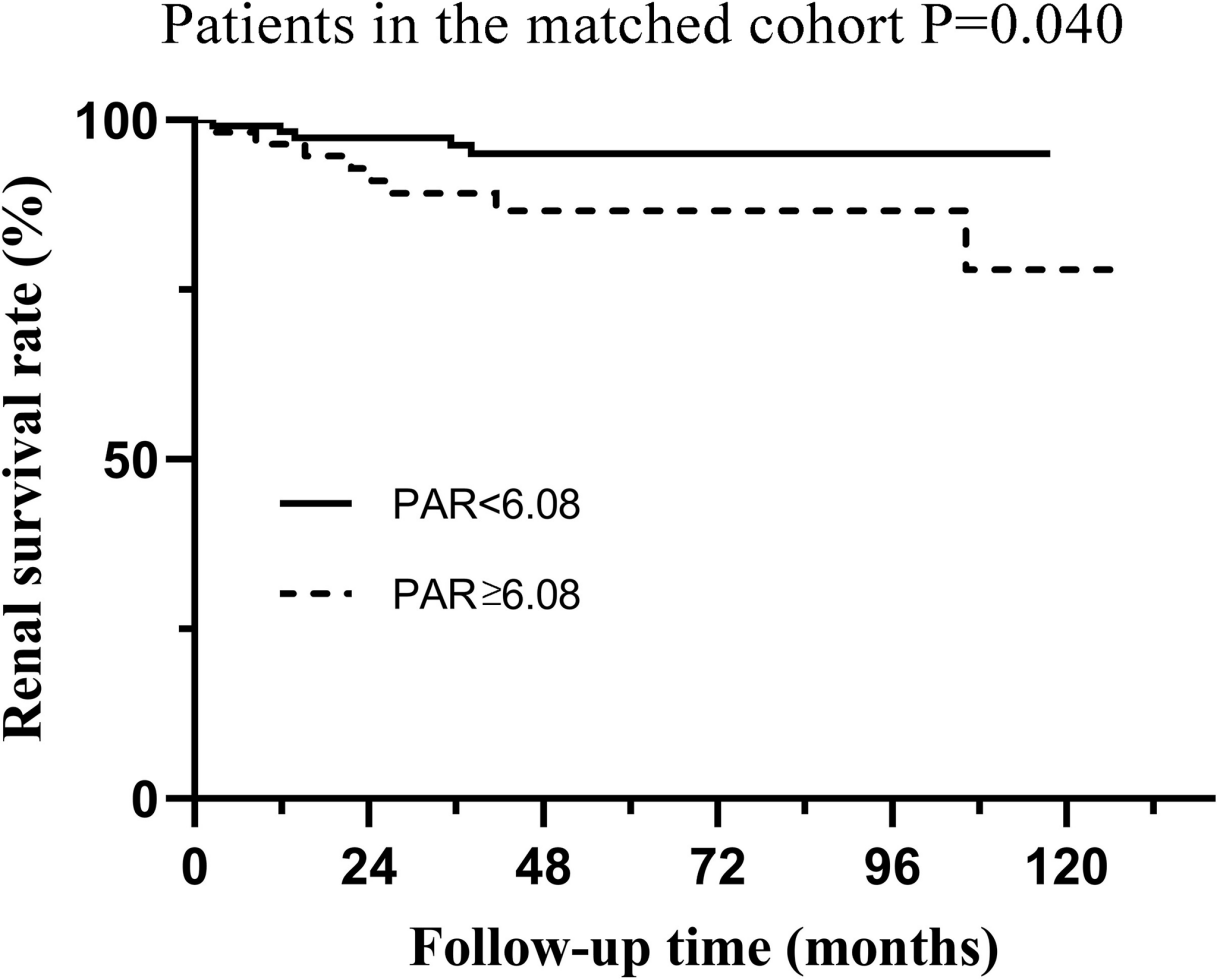
Kaplan-Meier analysis for the endpoint of ESRD in the matched cohort which was divided by PAR.

Although Cox regression analysis (**Table 1**) and Kaplan–Meier survival curves (**Figure 2**) suggested that PAR could serve as an independent prognostic marker of IgAN, propensity matching and subgroup analyses were also conducted to verify the effect of PAR in predicting renal prognosis. The unbalanced conditions at baseline between the two groups were regulated by PS matching to eliminate the influence of the correlation of PAR with clinicopathological manifestations. The 1:2 PS matching using the greedy matching algorithm obtained the matched pairs of 115 patients with low PAR and 57 patients with high PAR. Taken as a whole, it turned out that only PLT and ALB had significant differences in the two groups. The remaining elements were comparable in the matched cohort (**Table 3**). The Kaplan-Meier survival of the matched cohort further attested to the conclusion that the PAR could powerfully prognosticate renal outcomes. Similar to the nonmatching group, patients with low PAR had definitely higher renal survival rates than high PAR patients (p=0.004), which revealed that PAR had a fairly good predictive efficacy (**Figure 3**).

**Figure 4:**
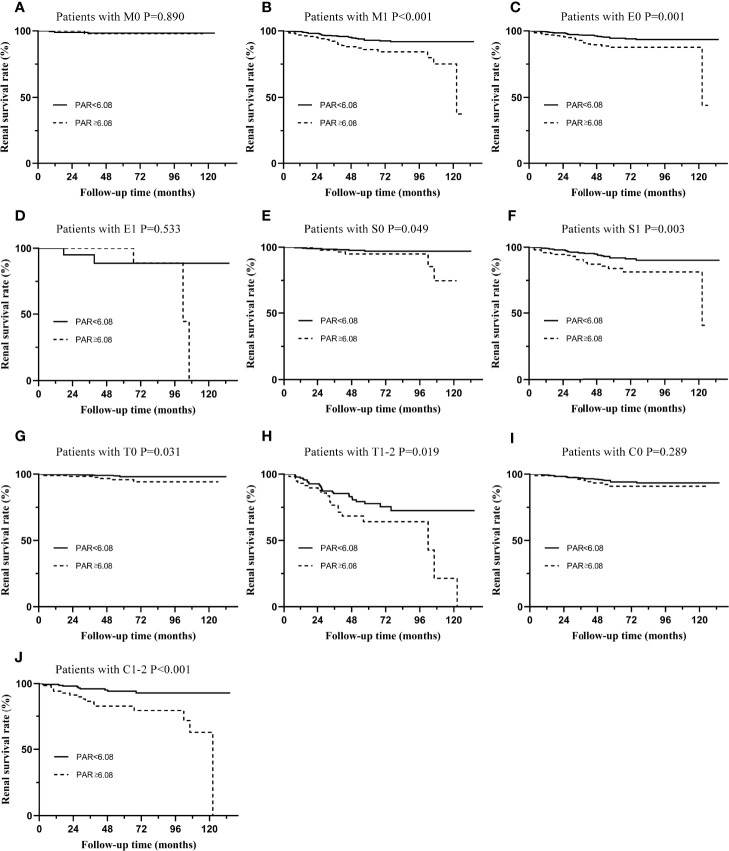
Subgroup analysis of IgAN patients with different types of pathological lesions. The Kaplan-Meier curve of patients with M0 **(A)**, M1 **(B)**, E0 **(C)**, E1 **(D)**, S0 **(E)**, S1 **(F)**, T0 **(G)**, T1-2 **(H)**, C0 **(I)**, C1-2 **(J)** in two groups distinguished by PAR.

**Figure 5:**
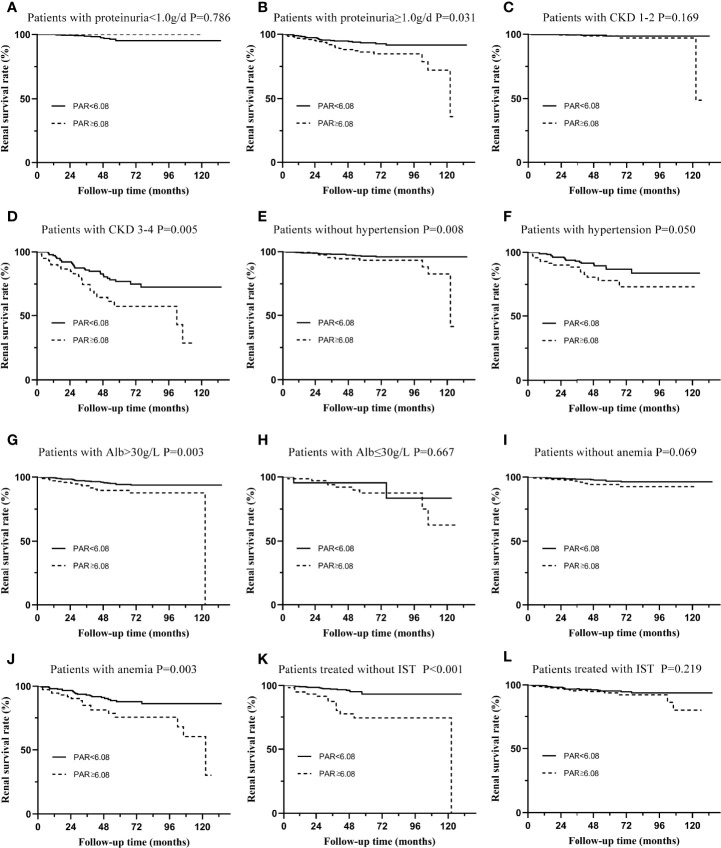
Subgroup analysis of IgAN patients with different types of clinical manifestations. **(A)** The renal survival curve of patients with proteinuria≤1.0g/d in two groups separated by PAR. **(B)** The renal survival curve of patients with proteinuria ≥1.0g/d in two groups separated by PAR. **(C)** The renal survival curve of patients with CKD 1-2 in two groups separated by PAR. **(D)** The renal survival curve of patients with CKD 3-4 in two groups separated by PAR. **(E)** The renal survival curve of patients without hypertension in two groups separated by PAR. **(F)** The renal survival curve of patients with hypertension in two groups separated by PAR. **(G)** The renal survival curve of patients with Alb>30g/L in two groups separated by PAR. **(H)** The renal survival curve of patients with Alb≤30g/L in two groups separated by PAR. **(I)** The renal survival curve of patients without anemia in two groups separated by PAR. **(J)** The renal survival curve of patients with anemia in two groups separated by PAR. **(K)** The renal survival curve of patients treated without IST in two groups separated by PAR. **(L)** The renal survival curve of patients treated with IST in two groups separated by PAR.

Subgroup analyses based on different pathological characteristics (**Figure 4**) and clinical manifestations (**Figure 5**) were then carried out. It should be noted that PAR might be an ideal parameter to identify the survival rate of IgAN patients with M1 (P=0.031, **Figure 4B**), E0 (P=0.001, **Figure 4C**), S0 (P=0.049, **Figure 4E**), S1 (P=0.003, **Figure 4F**), T0 (P=0.031, **Figure 4G**), T1-2 (P=0.019, **Figure 4H**) and C1-2 (P<0.001, **Figure 4J**). However, PAR might not seem to distinguish the prognosis of IgAN patients with M1, E1, and C0. Additionally, it was illustrated that PAR was a novel marker for ESRD in IgAN patients with proteinuria≥1.0 g/d (P=0.031, **Figure 5B**), CKD 3-4 (P=0.005, **Figure 5D**), normal blood pressure (P=0.008, **Figure 5E**), hypertension (P=0.050, **Figure 5F**), Alb>30 g/L (P=0.003, **Figure 5G**), and anemia (P=0.003, **Figure 5J**), showing that PAR was more suitable for forecasting in these situations. Our results also indicated that patients with high PAR usually had adverse renal survival when they did not receive any immunosuppressive therapy. However, for patients treated with immunosuppressive therapy, there were no significant disparities between the two groups (**Figures 5K, L**).

## Discussion

IgAN is a prevalent disease in which multiple factors are involved in its development and progression (13). Although the definite pathogenesis of IgAN is still unclear, most scholars agree that autoimmunity and inflammation play important roles in it (2, 13). In recent research, platelet-related parameters have been found to be related to chronic kidney disease (CKD), diabetic nephropathy, sclerosing nephropathy, and some other renal diseases (14, 15). Accordingly, it is supposed that platelets might be associated with IgAN.

Without considering the effect of covariates, the K-M curve found that PLT, PLR, and PAR were strongly correlated with the renal outcomes of IgAN (**Figure 2**), suggesting that all of them might serve as potential predictors. ROC curves (**Figure 1**) further demonstrated that PAR and PLR had better predictive power than PLT, where the predictive values of PAR and PLR were similar. Multivariate Cox regression adjusting for demographic data, pathological findings, treatment, and laboratory results indicated that compared with PLR, albumin, and PLT, PAR seemed to be a better marker of adverse renal outcome (**Tables 1**, **2**), implying PAR was the only platelet-related parameter that could be used as an independent risk factor. Although there was no report on the relationship between PAR and IgAN, PAR was proven to be closely related to the severity and prognosis of many inflammatory diseases. Yukai Wang et al. reported that PAR was positively correlated with the disease activity of axial spondyloarthritis (16). PAR was also illustrated to predict the mortality of patients suffering from severe fever with thrombocytopenia syndrome (17). PAR plays a similar role in predicting outcomes of patients with peritoneal dialysis (18). Hence, the consistent results of multiple articles suggested that the PAR might become a novel prognostic indicator in the future (4, 5, 16–18).

To more accurately evaluate the predictive power of the PAR, we analyzed the predictive effect of the PAR on patients with different clinicopathological characteristics through subgroup analysis. Grouped by Oxford classification, PAR tended to have great predictive power in patients with M1, E0, S0, S1, T0, T1-2, and C1-2, while it might lose its ability in patients with M0, E1, and C0. It should be noted that the majority of the enrolled patients had no pathological damage, such as mesangial hypercelluarity and endocapillary hypercellularity. In other words, the sample size of M0 and E1 patients was small, which might be an important reason for the negative conclusion. Undoubtedly, these statistical results need to be confirmed by further large-sample clinical cohorts. PAR could not predict the prognosis of C0 patients well because PAR might interact with the crescent; that is, patients with high PAR might have a higher risk of developing crescent. The interaction between the two was an important reason for the negative results. Similarly, grouped by the clinical manifestations, PAR was more suitable for the prediction of proteinuria≥1.0 g/d, CKD 3-4, normal blood pressure, hypertension, Alb>30 g/L, and anemia. Our results also indicate that patients with high PAR usually had adverse renal survival when they did not receive any immunosuppressive therapy. These results seemed to show that PAR might better predict the prognosis and outcome of patients who were already very severe, which might be because patients with mild renal impairment progressed to ESRD relatively slowly, while patients with severe renal impairment developed into the end -point relatively quickly (19).

At present, it is not completely clear why the PAR can predict the prognosis of IgAN. We initially thought that high PAR patients seemed to have severer clinical manifestations and pathological lesions since high-grade proteinuria, endocapillary proliferation, and crescents were more frequent in the high PAR group, which might explain the relationship between PAR and prognosis. Interestingly, after eliminating the influence of different baselines on outcome variables, PAR could still predict the poor prognosis of IgAN. Therefore, the previous explanation did not seem to fully explain this phenomenon. Emerging studies have revealed that the platelet count is positively related to some chronic inflammatory markers (20, 21). In addition to reflecting the nutritional status of the body, ALB is considered to be associated with inflammation (22, 23). It is proposed that a new indicator PAR that combines the above two might be able to better reflect the body’s inflammatory state (24). Chronic inflammation undoubtedly participated in the pathogenesis and development of IgAN, and inflammatory mediators might damage the structure and function of the kidney tissue (25). Hence, a high level of PAR, meaning a hyperinflammatory state and/or poor nutrition, definitely predicts the adverse renal outcomes of IgAN.

PAR, the PLT divided by the ALB, is easy to obtain in the clinic through peripheral white blood cell (WBC) count and liver/kidney function tests, and is much cheaper than other inflammatory indicators. In addition, PAR is a combination marker that seems to be more accurate in prediction than other platelet-related parameters. It has been reported that PAR might be more stable and less likely to be affected by dynamic physiological conditions than other platelet parameters and/or inflammatory markers (17, 18, 26, 27). As PAR seemed to be related to the inflammatory responses, it would be crucial to show whether PAR was correlated with one of the most used inflammatory serum markers such as the C-reactive protein (CRP). Clinically, at least in our medical center, CRP and IL-6 are not routine examination items for patients with IgA nephropathy, and there is no guideline recommending routine examination of CRP and IL-6 in IgAN patients. It should be noted that only 251 patients had CRP tests. There is a large amount of missing data, so there is a certain bias in the statistical analysis. Nevertheless, we performed Spearman correlation analysis and found that PAR and CRP were significantly positively correlated (r=0.612, P<0.001), implying that PAR is suggestive of inflammatory responses in IgAN. Besides, several studies have reported that PAR was indeed an inflammatory index, so it was believed that PAR might be a preferred one since it was cheap and easy to calculate in the clinic. Hence, PAR, a novel inflammation marker, might be used in clinical practice.

Previous studies have suggested proteinuria was a well-established risk factor for kidney function decline in IgAN. However, the proteinuria was not shown to be an independent risk factor for the renal outcomes. The following reasons may explain this phenomenon. Our study found that patients in the high PAR group usually presented with higher levels of proteinuria. At the same time, the correlation analysis suggested that there was a positive correlation between PAR and proteinuria (r=0.33, P<0.001). When these two indicators were put into the model at the same time, the predictive effect of these indicators might be masked, which might explain why the proteinuria was not shown to be an independent risk factor for the renal outcomes. Notably, PAR could still serve a good marker for prognosis, implying it was strongly correlated with the prognosis of IgAN. To demonstrate this, we excluded platelet-related indicators from this COX model and found that proteinuria indeed remained an independent risk factor for IgAN (HR 1.123, 95%CI 1.02, 1.230, P=0.013), which was consistent with our speculation. This seems to suggest that inflammation state might be better predictors of IgAN prognosis than proteinuria. But this speculation needs further verification.

Limitations should be noted in this study. First, this was a single-center study with a relatively short follow-up period. Second, the optimal cut-off value was based on this cohort, which means it might not be suitable for other populations and races. Third, since this was a retrospective study, there were no detailed data for regular follow-up in each subject, so dynamic analysis of PAR could not be performed. Fourth, retrospective analyses are prone to residual confounding effects of comorbidity, lift style, and medications that affect blood cell counts and selection bias. Therefore, high-quality prospective studies with large samples are required.

## Conclusion

PAR is an ideal inflammatory parameter to identify the renal outcomes of IgAN patients with severe clinicopathological manifestations and could be used as an independent risk factor for IgAN progression.

## Data Availability Statement

The original contributions presented in the study are included in the article/**Supplementary Material**. Further inquiries can be directed to the corresponding author.

## Ethics Statement

The study was approved by the Ethical Committee of West China Hospital of Sichuan University (2019-33). The patients/participants provided their written informed consent to participate in this study.

## Author Contributions

Study design: WQ, YT, and TJX. Data acquisition: WQ, JT, SW, ZJ, LD, GS, XL, and AQ. Data analysis: JT and GS. Supervision: WQ and YT. Each author accepted accountability for the overall work by ensuring that questions pertaining to the accuracy or integrity of any portion of the work are appropriately investigated and resolved. All authors contributed to the article and approved the submitted version.

## Funding

This study was partly supported by the National Key R&D Program of China (2020YFC2006503) and grants from the project of the National Natural Science Foundation of China (No. 81970612).

## Conflict of Interest

The authors declare that the research was conducted in the absence of any commercial or financial relationships that could be construed as a potential conflict of interest.

## Publisher’s Note

All claims expressed in this article are solely those of the authors and do not necessarily represent those of their affiliated organizations, or those of the publisher, the editors and the reviewers. Any product that may be evaluated in this article, or claim that may be made by its manufacturer, is not guaranteed or endorsed by the publisher.
