# Supplementary material for: Platelet-to-Albumin Ratio: A Novel IgA Nephropathy Prognosis Predictor
Source: Front Immunol. 2022 May 19;13:842362. doi: 10.3389/fimmu.2022.842362 (PMC9162245; doi:10.3389/fimmu.2022.842362)
Supplement: Supplementary file 1 [file DataSheet_1.docx]

| **Variables** | **Unmatched Cohort** | | | **Matched Cohort** | | |
| --- | --- | --- | --- | --- | --- | --- |
|  | **Low PAR group** | **High PAR group** | **P** | **Low PAR group** | **High PAR group** | **P** |
| **Numbers (%)** | 723 (74.8) | 243 (25.2) |  | 115 (66.9) | 57 (33.1) |  |
| **IgG** | 11.10 (9.22, 12.80) | 9.45 (6.26, 11.90) | **<0.001** | 11.10 (9.54, 12.30) | 9.59 (6.67, 11.65) | **0.003** |
| **IgA** | 2.87 (2.25, 3.55) | 2.76 (2.07, 3.61) | 0.352 | 3.07±1.02 | 2.86±1.01 | 0.190 |
| **IgM** | 1.25 (0.95,1.79) | 1.37 (0.90, 1.98) | **0.030** | 1.42±0.68 | 1.56±0.83 | 0.271 |
| **C3** | 0.89 (0.78, 1.01) | 0.90 (0.79, 1.01) | **0.048** | 0.9219±0.1919 | 0.95±0.24 | 0.401 |
| **C4** | 0.21±0.07 | 0.23±0.07 | **0.011** | 0.2147±0.0602 | 0.22±0.07 | 0.362 |

**Supplementary Table 1 Immunology related indicatorsof the IgAN patients at baseline, grouped by the platelet to albumin ratio.**

**Supplementary Table 2 The change of PAR after treatment of IgAN patients**

| Parameters | Treatment | The change value of PAR | P value |
| --- | --- | --- | --- |
| Low PAR group  n=723 | Support treatment | 0.20 (-0.82, 0.99) | 0.282 |
|  | Immunosuppressive treatment | 0.28 (-0.94, 1.12) |  |
| High PAR group  n=243 | Support treatment | -1.02 (-2.17, 0.01) | 0.173 |
|  | Immunosuppressive treatment | -0.77 (-1.81, 0.00) |  |
